# Development and implementation of telehealth for peritoneal dialysis and kidney transplant patients monitoring during the COVID-19 pandemic

**DOI:** 10.1590/2175-8239-JBN-2020-0137

**Published:** 2020-11-30

**Authors:** Luciane M Deboni, Eviline M V Neermann, Viviane Calice-Silva, Marina A Hanauer, Arnaldo Moreira, Andrei Ambrósio, Denise B Guterres, Marcos A Vieira, Fabiana B Nerbass

**Affiliations:** 1 Fundação Pró-Rim, Joinville, SC, Brasil.

**Keywords:** Renal Replacement Therapy, Teleorientation, Remote Consultation, Coronavirus Infections, Peritoneal Dialysis, Kidney Transplantation, Terapia de Substituição Renal, Teleorientação, Consulta Remota, Infecções por Coronavirus, Diálise Peritoneal, Transplante de Rim

## Abstract

The coronavirus (Sars-Cov-2) pandemic raised the need for social distance to reduce its spread. Chronic kidney disease patients on renal replacement therapy are especially susceptible to developing the most severe form of COVID-19, and, at the same time, require regular medical and multidisciplinary periodic follow-up. On an emergency basis, Brazil’s professional regulatory bodies authorized telehealth assistance, which made possible to migrate from face-to-face to distance appointments in health services across the country, when necessary. This article’s main objective is to describe the process of developing and implementing telehealth for monitoring renal transplant patients and patients on peritoneal dialysis during the COVID-19 pandemic.

## Resumo

A pandemia do coronavírus (Sars-CoV-2) trouxe a necessidade do isolamento social para a diminuição de sua propagação. Pacientes renais crônicos em terapia renal substitutiva são especialmente suscetíveis a desenvolverem a forma mais grave da covid-19, e, ao mesmo tempo, necessitam de acompanhamento médico e multidisciplinar regular com consultas periódicas. Em caráter emergencial, órgãos regulatórios profissionais brasileiros passaram a autorizar o atendimento da equipe de saúde por meio da teleconsulta, o que tornou possível a migração das consultas presenciais para consultas a distância nos serviços de saúde em todo o país, quando necessário. O principal objetivo deste artigo foi descrever o processo de desenvolvimento e implementação da teleorientação para acompanhamento de pacientes transplantados renais e em diálise peritoneal de um serviço de Joinville, SC, durante a pandemia de covid-19.

## Introduction

Covid-19 is an infectious disease caused by a newly discovered coronavirus (Sars-CoV-2) (December 2019). Most people infected with the virus will experience mild to moderate respiratory illness and will recover without the need for special treatment. Elderly people and those with underlying medical problems, such as cardiovascular disease, diabetes, chronic respiratory diseases and cancer, are more likely to develop the disease in a more severe way and, consequently, with a higher risk of death.^1^ Currently, elderly patients with such comorbidities are the majority of people with chronic kidney disease undergoing renal replacement therapies (RRT). In transplant recipients (Tx), due to the need for immunosuppression, symptoms and complications can be expected to be more intense, in addition to a longer time for virus spread - potentially increasing the risk of transmission to contacts, including healthcare professionals.^2^

In our service, located in the Southern Brazil until the beginning of the pandemic (March 2020), patients on peritoneal dialysis (n=140) and post-Tx (n=820) treatment were monitored in face-to-face appointments. Peritoneal dialysis (PD) patients came to the unit once a month. For post-Tx patients, the frequency ranged between twice a week, in the first post-Tx month, to once every two months, after completing the first year.

On March 17, 2020, the government of Santa Catarina state declared an emergency situation throughout the territory for the purpose of preventing and combating covid-19 (Decree nº 515).^3^ At the same time, with the objective of reducing the coronavirus spread in the country, professional regulatory agencies authorized the telehealth for distance monitoring. The letter from the Federal Board of Medicine No. 1756/2020, published on March 19, 2020,^4^ “recognizes the possibility and ethics of using telemedicine in an exceptional way and while the battle to fight the contagion of Covid-19 lasts”. The Federal Board of Nursing, through Resolution nº 634, of March 26, 2020, “authorizes and regulates Nursing Teleconsultation”.^5^ The same procedure was adopted by other healthcare professional boards.^6^^,^ 7

Thus, it became possible to conduct remote consultations on an emergency basis in healthcare services across the country, which is particularly relevant for patients on renal replacement therapy (RRT), for whom periodic monitoring by the healthcare team is essential to improve treatment compliance and effectiveness.

Thus, our goal was to describe the process of development and implementation of telehealth for monitoring renal transplant patients and those on peritoneal dialysis during the covid-19 pandemic in our service.

## Methods

This is a descriptive study of the development and implementation of telehealth for peritoneal dialysis and kidney transplant patients treated in a Nephrology center in Joinville, SC. Several departments participated in this process including the executive board, multidisciplinary team (doctors, nurses, dietitians, psychologists), quality, legal and information technology.

The action plan to setup the telehealth was divided among the departments involved with the following activities:

- The multidisciplinary team established the needs for telehealth implementation, including prescriptions, records in the patient's medical record, traceability and documentation of telephone contact, as well as the importance of a traceble recording of patient consent. Also, defined the criteria for patients selection for telehealth.

- The IT team studied alternatives for the development of a computerized record system, meeting the needs of the multidisciplinary team on the legal provisions of telehealth (such as date recording, start and end time, contact number, consent record and the means of communication used).

- The quality team developed the flowchart of the entire process to ensure that all necessary steps were carried out in a reproducible, traceable, subject to external audit. At the same time, patient safety, with the correct recording of information and standardization of care, meeting all the rules of the current legislation, were respected.

- Our legal department revised the recently published legislation, looking for new standards issued by regulatory agencies, enabling the computerized service, also complying with the General Law on Personal Data Protection (PDPL).^8^

## Results and Discussion

The multidisciplinary team defined that telehealth could be carried out as long as the professional had access to the patient’s electronic medical records, with the possibility of recording all actions taken during contact, as well as issuing prescriptions and reports, when necessary. Chart 1 describes the eligibility criteria for telehealth established by the healthcare team.

**Chart 1:** Eligibility criteria for telehealth

Transplanted kidney	1. Having completed more than three months of renal transplant;
	2. Be clinically stable in the past previous in-person visits (attending physician defines through medical chart review);
	3. Dwelling in places without the possibility of commuting to the healthcare facility;
	4. Consent with the teleconsultation*
Peritoneal dialysis	1. Patient reports being clinically well and with the adequate laboratory tests.*
	2. Consent with the teleconsultation*

* Checked by a telephone call from the nursing team before the scheduled consultation.

In addition to the inclusion criteria, those who reported Covid-19 symptoms during nurse contact were instructed to seek hospital care.

For both types of RRT, PD and post-Tx, we decided that the nurse would make an initial appointment, to check the patient’s health status and confirm the possibility of telehealth by the doctor. This facilitated and enabled the signs and symptoms presence that could lead to the suspicion of covid-19. In these cases, the patient would be advised on the necessary care and eventual search for hospital service. Those patients who reported signs or symptoms that would require face-to-face medical evaluation were scheduled at spaced times and with companion restrictions, respecting the rules from the Ministry of Health,^9^ in order minimize the movement of people and reduce the risk of contagion by covid-19.

Regarding the telehealth tool, the biggest doubt was regarding the technological means to be used. As many patients are elderly and several have deprived social conditions, we considered that videoconferencing would not be feasible for everyone. Therefore, we decided to use the telephone call. Although the Federal Board of Medicine had already authorized telemedicine, there were no regulations from the paying sources for this assistance. For this reason, there was a concern to create an effective documentation of the telehealth, which would be registered in the medical record and could be audited by the inspection and funding agencies. The solution found by the IT team was the recording of the calls by the digital telephone exchange linked to the extension used and the record of the extension number. Thus, if necessary, it is possible to revisit the recorded call and prove the service, always maintaining the security and confidentiality of the information. From instructions from the legal department and the rules of good clinical practice, at the time of the call, the patient is explained that it will be recorded and verbal consent is requested. Only after the patient`s consent the service is provided.

**Figure 1:**
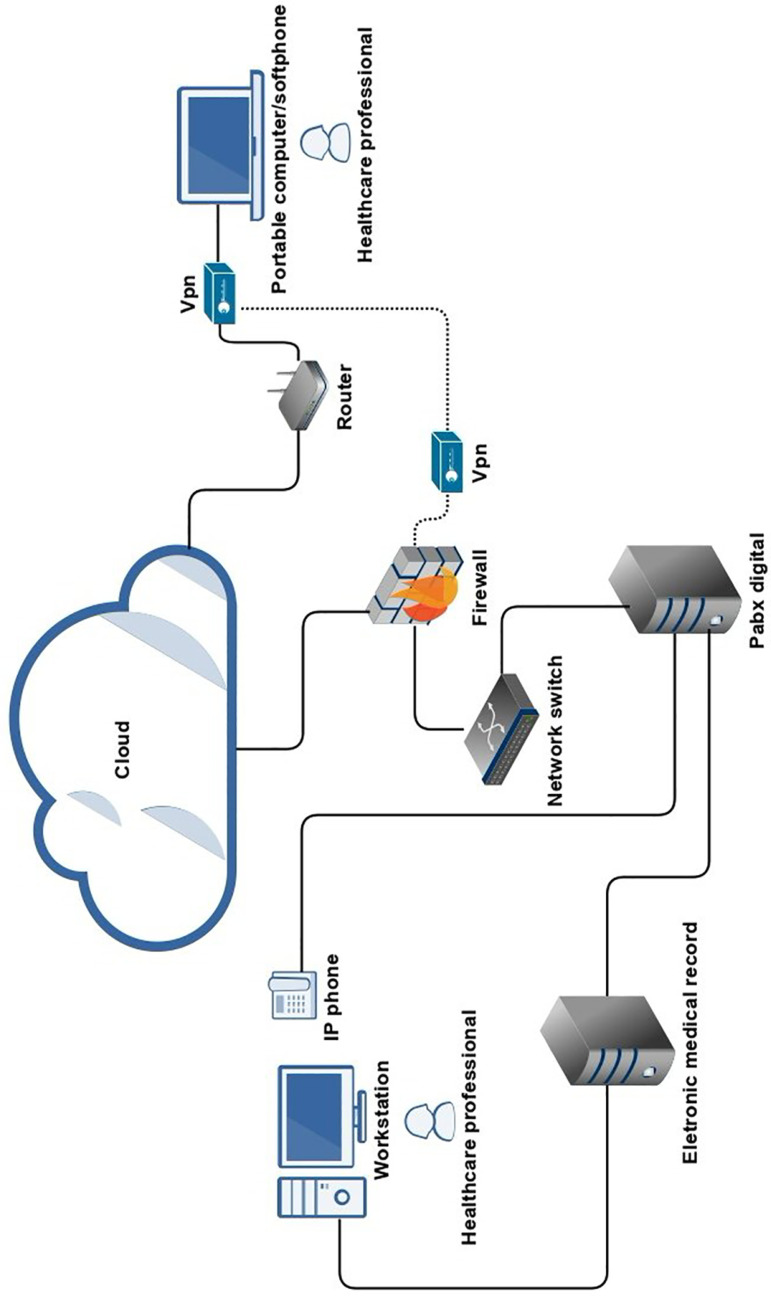
Technological resources used to implement remote care.

Legend: Provision of IP extensions (digital telephone system that uses the internet protocol) specific to provide care with all the features of integral recording of calls by means of a digital telephone exchange (Digital PABX). For professionals who were doing the work remotely, extensions were set up directly on the personal computer, using a multimedia softphone application. In order for this professional to access the IP telephony resource, a virtual private network connecting to the digital PABX Server, called VPN or “Virtual Private Network”, was set up. Access to the Clinical Hospital System (Tasy) was also made via VPN connection for professionals who were working remotely. In the Hospital Clinical System, classifications were created in the diaries to identify the phone calls.

Figure 1 shows the technological resources used to implement the telehealth.

Another concern was regarding the need for prescription generation. At first, if necessary, the doctor would leave the prescription with the reception at the clinic, available for collection by a family member. In April 2020, due to the national difficulty in issuing prescriptions in a virtual way, the Regional Board of Pharmacy and the Regional Board of Medicine developed a platform in electronic files with the professional’s digital signature. Thus, the use of this platform was included in the process. As an e-mail address is required to send the prescription to the patient and many of them do not have an e-mail address, a specific e-mail account for prescriptions was generated. The clinic receptionist receives by e-mail and forwards the file through a communication applicative to the patient. The implementation of the technological resources used for telehealth was possible in a short time because the institution already has structure and knowledge about these tools, used for some years by the call center department.

To standardize care and ensure that all necessary information would be recorded at the time of care (e.g. the patient’s phone number) and there would be a standardization of this type of care within the institution, we created a consultation model, standardized within the computerized system used in the institution (TASY®, Phillips).

**Figure 2:**
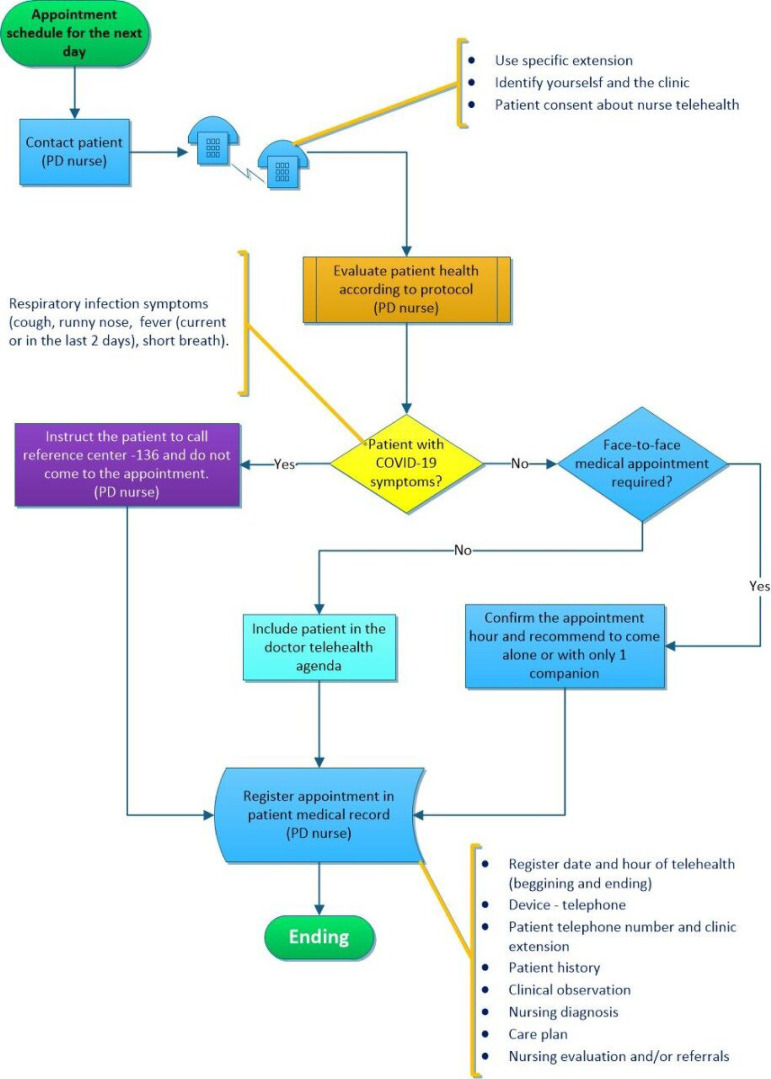
Flowchart of telehealth for peritoneal dialysis patients.

**Figure 3:**
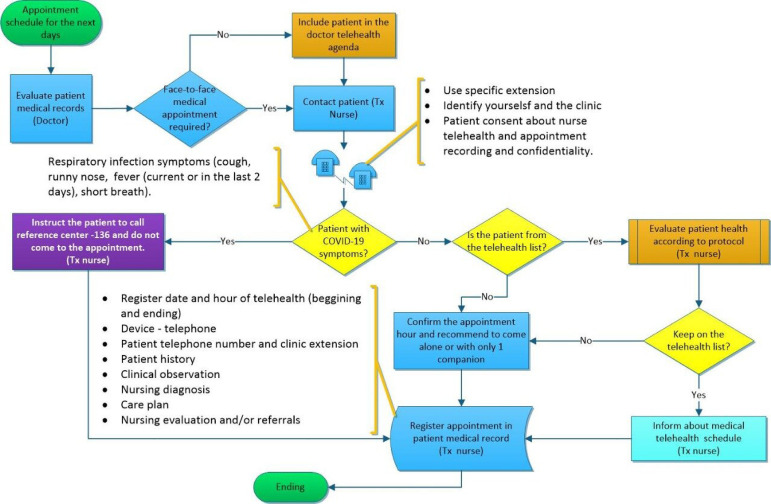
Flowchart of telehealth for kidney transplant patients.

In the end, a flowchart was developed by the quality department, following the guidelines and regulations in force (Figures 2 and 3).^4^^,^ 5 ^,^ 9 ^-^ 12

From the first calls, on April 14, 2020, there was good acceptance by patients. We also understood that it would be necessary to include the possibility of having remote care by a psychologist and dietitian, both under the authorization of the respective professional boards. Therefore, when the doctor perceive the necessity the multidisciplinary team is asked to schedule and appointment.

Telehealth made it possible to maintain the social isolation of many patients, encouraging at-risk group professionals to work from home, preserving the health of many who are at the forefront by decreasing the flow of people in the clinic environment.

## Conclusion

The coronavirus pandemic has brought on a new reality and great challenges, and humanity needs to adapt to new needs. The main way to contain the spread of viral contamination is social isolation. Chronic renal patients in RRT need regular medical and multidisciplinary periodic follow-up. The maintenance of this care model would result in an increased risk of contagion for these patients. The knowledge from the different departments of the institution (doctors, multidisciplinary team, information technology, quality control, infection and legal) enabled the creation of this tool for the record of remote consultation in a traceable way, which meets all the requirements of the regulatory agencies and paying sources. Also, it made it possible to maintain outpatient care for this group of patients, minimizing their risk of contagion.
